# Differences in e-cigarette use, dependence, and knowledge between health and social sciences university students in Costa Rica

**DOI:** 10.3389/froh.2026.1834758

**Published:** 2026-05-29

**Authors:** Noelia Solano-Maroto, Carolina Garro-Mata, Diana Gómez-Solano, Nicole Padilla-Fonseca, Cristina Barboza-Solís, Francisco Jiménez-Bolaños, Karol Ramírez

**Affiliations:** 1 Faculty of Dentistry, University of Costa Rica, Finca 3 “Instalaciones Deportivas”, Montes de Oca, San José, Costa Rica; 2 School of Statistics, University of Costa Rica, Montes de Oca, San José, Costa Rica

**Keywords:** behavior, e-cigarettes, knowledge, university students, vaping

## Abstract

**Introduction:**

The use of vapes or electronic cigarettes (e-cigarettes) has increased worldwide. However, studies evaluating their use among university students in Latin American countries remain scarce. Consequently, the level of knowledge that students have about these devices is not well understood. Moreover, emerging evidence suggests that e-cigarette use may be associated with periodontal diseases.

**Objectives:**

To estimate the prevalence and dependence of e-cigarette use, compare knowledge regarding these devices, and examine the association between e-cigarette use and self-perceived periodontal health among Health Sciences (HS) and Social Sciences (SS) students.

**Methods:**

An online cross-sectional questionnaire was conducted among HS and SS students aged 18–30 years, of the Rodrigo Facio Campus, University of Costa Rica.

**Results:**

A total of 771 students answered the questionnaire, 494 of HS and 277 of SS. Mean age of participants was 21,713(±2,780) years. The prevalence of e-cigarettes use was 9% in HS students and 22% in SS students (*p* < 0.001). Most e-cigarette users from both groups, reported having no dependence on e-cigarettes (*p* = 0.168). SS students who vaped, reported a greater medium dependence compared to HS (*p* = 0.039). No differences were found between groups on high dependence (*p* = 0.764). HS students were more informed about health risks associated with e-cigarettes (*p* < 0.05). HS students were more aware of toxic compounds commonly found in e-cigarettes, compared to SS students (*p* < 0.05). SS students are seeking information about e-cigarettes from less reliable sources (*p* < 0.001). HS students that vaped, observed changes in the color of their gums (*p* < 0.002). This was not perceived by SS students.

**Conclusions:**

Compared to SS students, HS students reported lower use and less medium dependence of e-cigarettes, were relatively more well-informed about health risks and harmful substances in e-cigarettes, sought information from more reliable sources, and those who vaped, perceived a detrimental change in periodontal health. It is important to provide university students with further education on vaping, including where to seek evidenced-based information of adverse effects.

## 1 Introduction

Vapes or electronic cigarettes (e-cigarettes) are battery-powered devices that generate aerosol when inhaled by users. They typically contain nicotine, flavorings, and other chemical substances (1). Although they may resemble conventional combustible cigarettes or appear in various other forms, their basic structure and operating mechanisms are similar (2). The concept of vaping devices dates back to 1965 (3), but early versions contained only flavorings without nicotine and therefore attracted little interest from tobacco companies or smokers (4). Their popularity increased in the early 2000s in the United States and Europe, when they were promoted as a potential smoking cessation aid and a less harmful alternative to conventional cigarettes (4–6).

However, current evidence indicates that vaping devices contain substances that may be harmful to both users and individuals exposed to secondhand aerosol (7). These include nicotine, flavoring agents, and heavy metals such as nickel, tin, lead, and chromium (8, 9). In addition, several compounds present in e-cigarette aerosols, including formaldehyde, acetaldehyde, and acrolein, have been classified as carcinogenic (10, 11). Although these devices may contain fewer toxic compounds than combustible cigarettes, they are not risk-free and may still pose significant health risks (3, 12). Consequently, the World Health Organization (WHO) states that there is insufficient scientific evidence to support the use of e-cigarettes as an effective smoking cessation therapy (13).

In recent years, there has been an increase in the use of vaping devices among adolescents and young adults. Unfortunately, there are few longitudinal studies evaluating the damage caused by vaporizers on oral health. In addition, existing studies vary widely in their methodologies. For example, some studies lack control groups. Most clinical research has consisted of observational studies, in which a direct cause-and-effect relationship cannot be established (3). This has led to discrepancies among findings and has limited the ability to draw firm conclusions. Despite these methodological limitations, available evidence suggests that vaping device use adversely affects general health, causing alterations across multiple body systems. Nevertheless, vaping devices have gained popularity in recent years. In addition, recreational use of these products has been observed among young university students.

In Costa Rica, the first importation of electronic cigarettes was recorded in 2007. However, the Ministry of Health did not begin investigating their consumption until 2015 (14). Evidence of increasing use emerged in the Sixth National Survey on Psychoactive Substance Use among Secondary School Students, conducted in 2021 by the Institute on Alcoholism and Pharmacodependency, which reported, that exposure to these devices among adolescents had tripled compared with the 2018 survey (15). In November 2023, the Costa Rican Social Security Fund reported the country's first confirmed case of vaping-associated pulmonary syndrome (16).

In response to growing public health concerns, Costa Rica has strengthened its regulatory framework of vaping products. Law 10066 on the Regulation of Electronic Nicotine Delivery Systems prohibits the use of these devices in public spaces and restricts their use among individuals under 18 years of age. More recently, the technical regulation RTCR 519:2025 established a binding sanitary standard for vaping liquids with and without nicotine. This regulation requires product notification to the Ministry of Health, prior laboratory analysis by the Costa Rican Institute for Research and Teaching in Nutrition and Health, before commercialization, and strict limits on nicotine concentration and chemical constituents. It also prohibits substances and flavorings considered attractive to minors, bans advertising, promotion, sponsorship, and online sales, mandates health warnings covering 50% of product packaging, and requires compliance with environmental regulations for product waste management.

Despite these important regulatory and public health measures aimed at addressing the risks associated with vaping products in the country, published evidence on vaping behaviors among university students in Costa Rica remains limited, highlighting the need for research that examines patterns of use, knowledge, and perceptions within this population. Therefore, the objective of this study was to determine the prevalence of vaping device use and related perceptions among undergraduate students in the health sciences and social sciences, aged 18–30 years, at the Rodrigo Facio campus of the University of de Costa Rica. Our hypothesis was that there are differences in the prevalence and perceptions of vaping device use, as well as in knowledge about these devices and their effects on general and oral health, between health sciences and social sciences university students.

## 2 Materials and methods

### 2.1 Participants and procedures

The present cross-sectional study was approved by the Scientific Ethics Committee of the University of Costa Rica (CEC-107-2024). Data were collected between April and August 2024 using an anonymous online questionnaire administered to undergraduate students from health sciences and social sciences careers.

The survey was distributed via Google Forms, ensuring complete anonymity. The online questionnaire was disseminated through social media using the official accounts of each student association. Faculty members from different academic areas distributed QR codes with the questionnaire link, as an invitation for students to participate in the study. This strategy enabled the recruitment of the required number of student participants through random participation, resulting in a representative sample. The provided link included a field in which participants were required to enter their institutional email address to filter verify that the information submitted corresponded to students enrolled at the University of Costa Rica.

Enrollment data for students aged 18 to 30 years in the health sciences and social sciences were obtained from the Registry and Information Office of the University of Costa Rica. This age range was selected because it represents the typical undergraduate population during the first academic semester of 2022.

The study population comprised two academic areas. The health sciences included students enrolled in medicine (*n* = 2,342), dentistry (*n* = 485), microbiology (*n* = 406), and pharmacy (*n* = 572). The social sciences included students from the Faculties of Social Sciences (*n* = 3,165), Law (*n* = 783), Economic Sciences (*n* = 4,230), and Education (*n* = 2,593). The total population across both areas was 14,576 students.

The sample size was calculated using a finite population correction formula:

$$n=\frac{N\cdot Z^{2}\cdot p\cdot (1-p)}{E^{2}\cdot (N-1)+Z^{2}\cdot p\cdot (1-p)}$$

where *N* *=* *14,576* (total population), *Z* *=* *1.96* (95% confidence level), *p* = 0.5 (assumed prevalence, selected to maximize sample size in the absence of prior estimates), and *E* *=* *0.05* (margin of error). Substituting these values, the calculated sample size was therefore 374.3, which was rounded up to 375 participants.

The sample was then proportionally allocated according to the distribution of students in each academic area, resulting in approximately 98 participants from health sciences and 277 from social sciences, ensuring adequate representation of both groups in the analysis.

### 2.2 Instrument, adaptation, validation, and pilot testing

The survey questionnaire was adapted from previously published studies (17–20). The instrument was developed in Spanish, using close-ended questions and consisted of four sections covering demographic variables, knowledge of the effects of vaping (17), use of vaping devices (17), the vaping dependency index (18, 19), and self-perception of periodontal health (20).

The adaptation process followed cross-cultural adaptation principles and involved both linguistic translation and content modification to ensure clarity, semantic equivalence, and cultural relevance for the target population. The sections addressing knowledge of vaping effects, use of vaping devices, and the vaping dependency index were primarily translated and linguistically adapted into Spanish, while the self-perception of periodontal health section underwent additional content modifications to improve its applicability to the study population. All items were reviewed by two experts, F.J.-B. and K.R., to ensure natural phrasing and conceptual equivalence with the original instruments.

The instrument was initially pilot-tested with 10 individuals who were not part of the study sample to evaluate the clarity, comprehensibility, and wording of the questions. Minor refinements were subsequently made based on participant feedback. Following this preliminary phase, the questionnaire was administered to 152 university students from different areas of study at the University of Costa Rica exclusively for instrument validation purposes. The responses obtained during this stage were not included in the final analyses of the present investigation and were used solely to evaluate and validate the questionnaire items. Internal consistency was assessed for the different sections of the questionnaire where applicable.

#### 2.2.1 Demographic variables

The sociodemographic variables that were considered were: age, gender, province, field of study, and year of study.

#### 2.2.2 Knowledge of the study population regarding the effects of vaping devices

The questions included in this section were adapted from McLeish et al. (17). This section of the questionnaire used a multiple-choice format, allowing participants to select more than one response option or the “Do not know/No response” option when applicable.

Participants were asked about their knowledge of the health risks associated with vaping devices, the ingredients contained in these products, and the sources they would consult for information regarding vaping devices. The specific questions were:

(1) “What are some of the negative health effects associated with the use of vaping devices? Select all that apply”. Participants possible answers were: (0) Increased risk of heart attack; (1) Stroke and coronary artery disease; (2) Increased risk of seizures; (3) Increased risk of depression; (4) Increased risk of lung diseases; (5) There are no significant negative health effects; (6) Do not know/No response. Participants could select the “Do not know/No response” option if they were unaware of the possible effects of vaping devices. If this option was selected, no additional responses could be chosen.
(2) “Which of the following ingredients are found in vaping devices? Select all that apply”: (0) Formaldehyde; (1) Volatile organic compounds; (2) Heavy metals (such as tin, lead, nickel, cadmium); (3) Benzoic acid; (4) Propylene glycol; (5) Do not know/No response. Participants could select the “Do not know/No response” option if they were unaware of the ingredients contained in vaping devices. If this option was selected, no additional responses could be chosen.
(3) “If you had questions or doubts about vaping devices, whom would you consult?”: (0) A friend; (1) Vape shop staff; (2) Google/Internet; (3) A dentist or other healthcare professional; (4) Other.

Internal consistency for this section was evaluated using Cronbach's alpha coefficient based on the pilot-testing responses obtained from the 152 university students who participated exclusively in the validation phase of the instrument. Prior to analysis, responses were numerically coded to permit statistical evaluation. The obtained Cronbach's alpha coefficient was 0.631, indicating moderate internal consistency. This result should be interpreted with caution, as the section included a limited number of items that addressed conceptually distinct aspects of vaping-related knowledge, including adverse health effects, product ingredients, and information-seeking behaviors. Consequently, a high inter-item correlation was not necessarily expected. In addition, the heterogeneous structure of the items and the use of multiple-response formats limited the suitability of treating these variables as a unidimensional scale. Therefore, Cronbach's alpha was considered an exploratory indicator of internal consistency rather than evidence of a psychometrically validated construct. This limitation may introduce some degree of misclassification and restrict the formal assessment of construct validity.

#### 2.2.3 Use of vaping devices

Participants were asked about their use of vaping devices with the following questions:

(4) “Have you ever used a vaping device?”
  1. Yes
  2. No
(5) “Do you currently use a vaping device?”
  1. Yes
  2. No

If the participant selected “No,” the subsequent questions were not administered, and the questionnaire was concluded.

(6) “How long have you been using vaping devices?”
  1. 1–6 months
  2. 6 months–1 year
  3. More than 1 year

Internal consistency analyses, including Cronbach's alpha, were not performed for this section because the items were intended to capture independent behavioral characteristics related to vaping status and duration of use rather than components of a single latent construct. Consequently, these variables were analyzed descriptively and individually, and the assessment of internal consistency was not considered appropriate.

#### 2.2.4 Vaping dependency Index

To assess dependence on electronic cigarettes among the study population, the Penn State Electronic Cigarette Dependence Index (ECDI), Spanish version validated in Colombia, was used (18, 19). Its use in the present study was supported by the linguistic and cultural similarities between Colombia and Costa Rica, as well as the shared Spanish language, which allowed its application without major adaptation. Although formal validation in other Spanish-speaking countries remains limited, the ECDI has been translated into Spanish and applied in diverse populations, with previous studies supporting its construct validity across different settings. In addition, the ECDI provides a brief and standardized measure of e-cigarette dependence, making it suitable for use in university populations.

The questionnaire consisted of 10 items evaluating frequency of use, time to first use after waking, nocturnal use, cravings, difficulty refraining from use, withdrawal-related symptoms, and perceived difficulty quitting. Each item was scored according to the original ECDI scoring system, and total scores were calculated by summing individual item scores.

The questionnaire included the following items and scoring criteria:

1. “How many cigarettes [times] per day do you usually smoke [use your electronic cigarette]?” [(assume that one “time” consists of around 15 puffs or lasts around 10 min)]
  Scoring: 0–4 times/day = 0; 5–9 = 1; 10–14 = 2; 15–19 = 3; 20–29 = 4; 30+ = 5.
2. “On days that you can smoke [use your electronic cigarette] freely, how soon after you wake up do you smoke your first cigarette of the day [first use your electronic cigarette]?”
  Scoring: 0–5 mi*n* = 5; 6–15 min = 4; 16–30 min = 3; 31–60 min = 2; 61–120 min = 1; 121 + min = 0.
3. “Do you sometimes awaken at night to have a cigarette [use your electronic cigarette]?”
  Scoring: Yes = 1; No = 0.
4. “If yes, how many nights per week do you typically awaken to smoke [use your electronic cigarette]?”
  Scoring: 0–1 nights = 0; 2–3 nights = 1; 4 + nights = 2.
5. “Do you smoke [use an electronic cigarette] now because it is really hard to quit?”
  Scoring: Yes = 1; No = 0.
6. “Do you ever have strong cravings to smoke [use an electronic cigarette]?”
  Scoring: Yes = 1; No = 0.
7. “Over the past week, how strong have the urges to smoke [use an electronic cigarette] been?”
  Scoring: None/Slight = 0; Moderate/Strong = 1; Very Strong/Extremely Strong = 2.
8. “Is it hard to keep from smoking [using an electronic cigarette] in places where you are not supposed to?”
  Scoring: Yes = 1; No = 0.

When participants had not used tobacco [an electronic cigarette] for a period of time or had attempted to stop using it, they were also asked:

9. “Did you feel more irritable because you couldn't smoke [use an electronic cigarette]?”
  Scoring: Yes = 1; No = 0.
10. “Did you feel nervous, restless, or anxious because you couldn't smoke [use an electronic cigarette]?”
  Scoring: Yes = 1; No = 0.

Total ECDI scores were categorized as follows: 0–3 = not dependent; 4–8 = low dependence; 9–12 = moderate dependence; and ≥13 = high dependence.

During both the pilot-testing phase and the final study, the ECDI section was administered exclusively to participants who reported current e-cigarette use, consistent with the intended purpose of the instrument to assess dependence among active users. In the pilot study, conducted with 152 participants, only 16 individuals reported current vaping and completed the ECDI. Internal consistency reliability was evaluated using Cronbach's alpha, which yielded a coefficient of 0.70 for the 10-item scale. This value indicates acceptable internal consistency for an exploratory study and supports the reliability of the Spanish-language ECDI for assessing e-cigarette dependence in this university population.

#### 2.2.5 Self-perception of periodontal health

Self-perceived periodontal health was assessed using dichotomous (yes/no) questions designed to evaluate participants’ perceptions of their gingival health. The instrument was based on the Self-Perceived Periodontal Health Questionnaire developed and validated by Saka-Herrán in Spain in 2020 (20). The original instrument consisted of 13 items with yes/no response options. The original questionnaire included the following items: “do you have periodontal disease?”; “do you think you might have gum disease”; “have you ever been diagnosed by a dental professional with periodontal disease or pyorrhea?”; “have you ever been told by a dental professional that you have lost bone around your teeth or that you have deep pockets?”; “in the last years have you noticed that some of your teeth move or are looser than normal?”; “in the past years have you noticed that your teeth are longer or that you have receding gums?”; “in the last years have you noticed that you see the roots of several of your teeth?”; “have you felt pain in your gums during the last months?”; “do you frequently use a stick or interproximal brush to clean your teeth?”; “have you ever visited a periodontist or a specialist in gum disease to treat gum disease?”; “have you ever had treatment for gum disease such as scaling or root planing?”; “have you lost teeth in recent years because of mobility?”; and “do your gums usually bleed either when brushing or chewing?”.

The questionnaire was adapted for the present study by K.R. and F.J-B., both specialists in Periodontology. Several items from the original instrument were excluded because they assessed manifestations of moderate and severe periodontal disease that were considered unlikely in the target population of university students aged 18–30 years. Specifically, items related to tooth mobility, gingival recession, visible root exposure, tooth loss due to mobility, previous periodontal treatment, specialist periodontal care, and professional diagnosis of periodontal disease or bone loss were removed because these conditions are more commonly associated with moderate-to-severe periodontitis and older populations. The adaptation aimed to improve the clinical relevance and discriminatory capacity of the instrument for detecting early signs and symptoms of gingivitis in a younger population.

The final adapted instrument retained the following items: “do you think you may have gum disease?”; “have you felt pain in your gums during the last months?”; “in recent months, have you noticed a change in the color of your gums?”; “in recent months, have you noticed your gums becoming enlarged or swollen?”; “do you frequently use dental floss?”; “do your gums usually bleed when using dental floss?”; “do your gums usually bleed when brushing your teeth?”; and “do your gums usually bleed when chewing?”.

After pilot-testing with 152 participants, the adapted instrument demonstrated an acceptable internal consistency, with an overall Cronbach's alpha coefficient of 0.70. Item-total correlation analysis showed that items related to perceived gum disease, gingival color changes, and gingival bleeding contributed most strongly to the reliability of the questionnaire, indicating an adequate discriminatory capacity between different levels of self-perceived periodontal health. In contrast, items related to gum pain, dental floss use, and gingival enlargement/swelling showed lower discriminatory performance compared with the remaining items. Although the questionnaire relied on dichotomous (yes/no) response options, which may reduce sensitivity and limit the ability to capture gradations in symptom severity or frequency, this format was maintained to preserve consistency with the original validated instrument and to facilitate comprehension and completion in the study population. Nevertheless, the adapted questionnaire demonstrated acceptable psychometric properties for exploratory use in this population of university students.

### 2.3 Statistical analysis

Statistical analyses were conducted using R Studio (version 4.4.1). Differences in self-perceived periodontal health, knowledge, and vaping dependence were assessed using the chi-square test with a significance level of 5%.

Logistic regression analyses were performed to evaluate the association between academic area and knowledge regarding the ingredients present in vaping devices, the negative effects associated with vaping, and the number of vaping devices by academic area, adjusting for potential confounding variables including sex, age, and academic year. Odds ratios (ORs) with their corresponding 95% confidence intervals (95% CIs) were estimated. A *p*-value < 0.05 was considered statistically significant.

The strength of association between students’ self-perceived periodontal health and vaping dependence was evaluated using Cramér’s V.

## 3 Results

### 3.1 Demographic characteristics of participants

Table 1 presents the sociodemographic characteristics of the participants. The minimum required sample size for representativeness was 375 students (98 from health sciences and 277 from social sciences). However, 494 responses were obtained from Health sciences students. Therefore, all collected responses were included in the analyses to maximize the use of available data.

**Table 1:** Demographic characteristics and prevalence of e-cigarettes use in health sciences and social sciences students, University of Costa Rica.

Parameter	Variable	Health Sciences	Social Sciences	Total	*p-*value*
		*n* = 494	*n* = 277	*N* = 771	
Gender	Male	135 (27%)	114 (41%)	249	***<0*** ***.*** ***001***
	Female	356 (72%)	153 (55%)	509	
	Other	3 (1%)	10 (4%)	13	
Age	**Mean**	22.121	20.985	21.713	
	**SD**	2.730	2.723	2.780	
E-cigarette					
use		46 (9%)	62 (22%)	108	***<0*** ***.*** ***001***
Academic year	First	29 (6%)	76 (27%)	105	***<0*** ***.*** ***001***
	Second	86 (18%)	56 (20%)	142	
	Third	125 (25%)	65 (24%)	190	
	Fourth	110 (22%)	55 (20%)	165	
	Fifth	120 (24)	17 (6%)	137	
	Sixth	24 (5%)	8 (3%)	32	

SD, standard deviation; *Chi-squared test;.

*p* values in bold denote statistical significance (*p* < 0.05).

A total of 771 university students participated in the study, and all completed the questionnaire in full, as responses to all items were required prior to submission; therefore, no incomplete questionnaires or missing data were recorded. In health sciences, participants included 135 men, 356 women, and 3 individuals who identified as another gender. In social sciences, 114 men, 153 women, and 10 individuals identified as another gender participated. Overall, women represented most of the sample (66%), followed by men (32%) and individuals of other genders (2%).

The mean age of the total sample was 21.71 ± 2.78 years. The mean age was 22.12 ± 2.73 years in the health sciences area and 20.99 ± 2.72 years in social sciences area.

Regarding academic year, among health sciences students, 6% were in the first year, 18% in the second year, 25% in the third year, 22% in the fourth year, 24% in the fifth year, and 5% in the sixth year. In social sciences, 27% were in the first year, 20% in the second year, 24% in the third year, 20% in the fourth year, 6% in the fifth year, and 3% in the sixth year. Third-year students showed the highest participation in both academic areas. A statistically significant difference was observed in academic year distribution between health sciences and social sciences students (*p* < 0.001).

Nine percent (*n* = 46) of health sciences students and 22% (*n* = 62) of social sciences students reported current vaping at the time the questionnaire was administered. The difference in vaping prevalence between the two groups was significant *(p* < 0.001), indicating a higher frequency of vaping among students from the social sciences area.

Finally, most students from both academic areas reported residing in the province of San José (*p* = 0.017), consistent with the administration of the questionnaire at the Rodrigo Facio campus, data not shown.

### 3.2 Use of e-cigarettes

Table 2 presents the distribution of participants who reported current vaping according to academic year. Among students from the health sciences who reported vaping, the highest proportions were observed in the third year (*n* = 13; 28%) and fifth year (*n* = 13; 28%). Additionally, 6 students (13%) were in the first year, 5 (11%) in the second year, 5 (11%) in the fourth year, and 4 (9%) in the sixth year.

**Table 2:** Prevalence of e-cigarette use by academic area.

Academic Year	Health Sciences *n* = 46	Social Sciences *n* = 62	Total *N* = 108
First	6 (13%)	18 (29%)	24
Second	5 (11%)	19 (31%)	24
Third	13 (28%)	12 (19%)	25
Fourth	5 (11%)	9 (14%)	14
Fifth	13 (28%)	3 (5%)	16
Sixth	4 (9%)	1 (2%)	5

Adjusted logistic regression analyses controlling for sex, age, and academic year were additionally performed; detailed adjusted results are not shown for brevity.

Among students from the social sciences who reported vaping, the highest proportions were observed in the second year (*n* = 19; 31%) and first year (*n* = 18; 29%), followed by the third year (*n* = 12; 19%). In addition, 9 students (14%) were in the fourth year, 3 (5%) in the fifth year, and 1 (2%) in the sixth year.

An adjusted logistic regression analysis controlling for sex, age, and academic year was performed to evaluate the association between academic area and current vaping status. Students from the social sciences showed significantly higher odds of reporting current vaping compared with students from the health sciences (OR = 2.36; 95% CI: 1.50–3.74; *p* < 0.001), data not shown for brevity.

### 3.3 Knowledge of e-cigarette ingredients

Table 3 presents the comparative analysis between academic areas regarding knowledge of vaping device ingredients. For benzoic acid, 62 (12.6%) Health sciences students correctly identified this ingredient as being present in vaping devices, whereas 432 (87.4%) reported not knowing. In the social sciences group, 17 (6.1%) students recognized the presence of benzoic acid, while 260 (93.9%) were unaware of it. The same interpretative approach was applied to the remaining components.

**Table 3:** Knowledge of e-cigarette ingredients by area of study.

Ingredients	Health Sciences		Social Sciences		
	Yes	No	Yes	No	*p*-value*
Benzoic Acid	62 (12.6%)	432 (87.4%)	17 (6.1%)	260 (93.9%)	***0*** ***.*** ***007***
Formaldehyde	104 (21.1%)	390 (78.9%)	25 (9%)	252 (91%)	***<0*** ***.*** ***001***
Propylene Gycol	117 (23.7%)	377 (76.3%)	27 (9.7%)	250 (90.3%)	***<0*** ***.*** ***001***
Volatile organic compunds	163 (33%)	331 (67%)	19 (6.9%)	258 (93.1%)	***<0*** ***.*** ***001***
Heavy metals	163 (33%)	331 (67%)	59 (21.3%)	218 (78.7%)	***0*** ***.*** ***004***
Don't know/ Didn't answer	265 (53.6%)	229 (46.4%)	199 (71.8%)	78 (28.2%)	***0*** ***.*** ***002***

*Chi-squared test; *p* values in bold denote statistical significance (*p* < 0.05).

Adjusted logistic regression analyses controlling for sex, age, and academic year showed that differences between academic areas remained statistically significant for formaldehyde, propylene glycol, volatile organic compounds, heavy metals, benzoic acid, and the “Don't know/Didn't answer” category; detailed adjusted results are not shown for brevity.

Overall, health sciences students demonstrated greater awareness of the components contained in vaping devices compared with social sciences students (all *Ps* < 0.05). Among Health sciences students, the most frequently identified ingredients were volatile organic compounds (*n* = 163; 33.0%) and heavy metals (*n* = 163; 33.0%), followed by propylene glycol (*n* = 117; 23.7%), formaldehyde (*n* = 104; 21.1%), and benzoic acid (*n* = 62; 12.6%). Additionally, 265 students (53.6%) selected the “Do not know/No response” option. In contrast, among social sciences students, 59 (21.3%) identified heavy metals, 27 (9.7%) propylene glycol, 25 (9.0%) formaldehyde, 19 (6.9%) volatile organic compounds, and 17 (6.1%) benzoic acid. A higher proportion of social sciences students selected “Do not know/No response” (*n* = 199; 71.8%). All differences between academic areas were statistically significant (all *Ps* < 0.05).

To evaluate whether the observed differences between academic areas remained after controlling for potential confounding variables, adjusted logistic regression analyses were performed controlling for sex, age, and academic year. Compared with students from the health sciences, students from the social sciences showed lower odds of correctly identifying formaldehyde (OR = 0.42; 95% CI: 0.25–0.67; *p* < 0.001), propylene glycol (OR = 0.30; 95% CI: 0.18–0.49; *p* < 0.001), volatile organic compounds (OR = 0.16; 95% CI: 0.09–0.26; *p* < 0.001), heavy metals (OR = 0.47; 95% CI: 0.32–0.69; *p* < 0.001), and benzoic acid (OR = 0.51; 95% CI: 0.27–0.90; *p* = 0.025) as ingredients present in vaping devices. In contrast, students from the social sciences showed higher odds of selecting the response option “Do not know/No response” (OR = 2.59; 95% CI: 1.83–3.70; *p* < 0.001). Overall, these findings indicate that the differences in knowledge regarding vaping device ingredients between the two academic areas remained significant even after adjustment for demographic and academic variables (data not shown).

### 3.4 Knowledge of health risks associated with e-cigarette use

Table 4 presents participants’ perceptions of the health risks associated with e-cigarette use, comparing responses across academic areas. Among health sciences students, the most frequently reported perceived risks were increased risk of heart attack (*n* = 329; 66.6%), stroke and coronary artery disease (*n* = 291; 58.9%), depression (*n* = 172; 34.8%), and seizures (*n* = 147; 29.8%). A small proportion selected “Do not know/No response” (*n* = 21; 4.3%). In contrast, social sciences students reported lower percentages for each of these risks: increased risk of heart attack (*n* = 140; 50.5%), stroke and coronary artery disease (*n* = 102; 36.8%), depression (*n* = 65; 23.5%), and seizures (*n* = 49; 17.7%). A higher proportion of social sciences students selected “Do not know/No response” (*n* = 30; 10.8%). All these differences between academic areas were statistically significant (*p* < 0.05). Regarding the statement “There are no negative health effects,” 12 (2.4%) health sciences students and 5 (1.8%) social sciences students selected this option. Similarly, for “Increased risk of lung diseases,” 472 (95.5%) health sciences students and 251 (90.6%) social sciences students endorsed this response. No statistically significant differences were observed between the groups for these two items.

**Table 4:** Knowledge of health risks associated with e-cigarette use by area of study.

Negative Health Effects of Using E-cigarrettes	Health Sciences		Social Sciences		
	Yes	No	Yes	No	*p* value*
There aren't any significant negative health effects	12 (2.4%)	482 (97.6%)	5 (1.8%)	272 (98.2%)	0.576
Don't know/Didn't answer	21 (4.3%)	473 (95.7%)	30 (10.8%)	247 (89.2%)	***<0*** ***.*** ***001***
Increased risk for seizures	147 (29.8%)	347 (70.2%)	49 (17.7%)	228 (82.3%)	**0** **.** **001**
Increased risk for depression	172 (34.8%)	322 (65.2%)	65 (23.5%)	212 (76.5%)	**0** **.** **006**
Increased risk for stroke and coronary diseases	291 (58.9%)	203 (41.1%)	102 (36.8%)	175 (63.2%)	***<0*** ***.*** ***001***
Increased risk for heart attack	329 (66.6%)	165 (33.4%)	140 (50.5%)	137 (49.5%)	**0** **.** **006**
Increased risk for lung disease	472 (95.5%)	22 (4.5%)	251 (90.6%)	26 (9.4%)	0.497

* Chi-squared test; *p* values in bold denote statistical significance (*p* < 0.05).

For increased risk of seizures, 147 (29.8%) Health sciences students reported being aware of this potential effect, whereas 347 (70.2%) indicated they were unaware of it. In the social sciences group, 49 (17.7%) students recognized that vaping devices may cause this health effect, while 228 (82.3%) reported not knowing. The same interpretative approach was applied to the remaining reported health effects.

Adjusted logistic regression analyses controlling for sex, age, and academic year were also performed. The main associations remained statistically significant after adjustment; detailed regression results are not shown for brevity.

Compared with students from the health sciences, students from the social sciences showed lower odds of correctly recognizing several negative effects associated with vaping, including increased risk of seizures (OR = 0.61; 95% CI: 0.41–0.90; *p* = 0.013), stroke and coronary artery disease (OR = 0.53; 95% CI: 0.38–0.73; *p* < 0.001), depression (OR = 0.61; 95% CI: 0.42–0.87; *p* = 0.007), and heart attack (OR = 0.67; 95% CI: 0.48–0.93; *p* = 0.016). Likewise, students from the social sciences showed higher odds of selecting the response option “Do not know/No response” (OR = 2.16; 95% CI: 1.13–4.20; *p* = 0.021). These findings suggest that the differences in knowledge regarding the negative effects associated with vaping between both academic areas remained significant even after adjustment for demographic and academic variables. Detailed regression results are not shown for brevity.

### 3.5 Penn state electronic cigarette dependence index

Table 5 presents the levels of vaping dependence among students from the health sciences and social sciences who reported using e-cigarettes. An equal number of students in both groups (*n* = 24 each) reported no dependence. No statistically significant differences were observed between groups for low or high levels of dependence. However, a significantly higher proportion of social sciences students reported moderate dependence (*n* = 15; 24.2%) compared with health sciences students (*n* = 4; 8.7%) (*p* = 0.039).

**Table 5:** Dependence on e-cigarettes among users by area of study.

Rating of dependence	Health Sciences *n* = 46	Social Sciences *n* = 62	*p* value*
No dependence	24 (52.2%)	24 (38.7%)	0.168
Low dependence	15 (32.6%)	18 (29.0%)	0.692
Medium dependence	4 (8.7%)	15 (24.2%)	**0** **.** **039**
High dependence	3 (6.5%)	5 (8.1%)	0.764

*Chi-squared test; *p* values in bold denote statistical significance (*p* < 0.05).

### 3.6 Source of e-cigarette information

Table 6 presents participants’ responses regarding whom they would consult if they had questions about vaping devices. Statistically significant differences were observed between academic areas for all sources of information (all *Ps* < 0.05). Among health sciences students, the most frequently selected source was Google/Internet (*n* = 296; 59.9%), followed by a dentist/healthcare professional (*n* = 208; 42.1%), vape shop staff (*n* = 84; 17.0%), and a friend (*n* = 72; 14.6%). In contrast, social sciences students most frequently reported consulting Google/Internet (*n* = 199; 71.8%), followed by a dentist/healthcare professional (*n* = 101; 36.5%), a friend (*n* = 67; 24.2%), and vape shop staff (*n* = 63; 22.7%).

**Table 6:** Number and percentage of participants divided by area of study, who chose each source of e-cigarette information.

Source for questions on e-cigarettes	Health Sciences		Social Sciences		
	Yes	No	Yes	No	*p* value*
Google/ Internet	296 (59.9%)	198 (40.1%)	199 (71.8%)	78 (28.2%)	***<0*** ***.*** ***001***
Dentist/ Medical doctor/ Health professional	208 (42.1%)	286 (57.9%)	101 (36.5%)	176 (63.5%)	***<0*** ***.*** ***001***
Friend	72 (14.6%)	422 (85.4%)	67 (24.2%)	210 (75.8%)	***<0*** ***.*** ***001***
Vape shop employee	84 (17.0%)	410 (83.0%)	63 (22.7%)	214 (77.3%)	***<0*** ***.*** ***001***

*Chi-squared test; *p* values in bold denote statistical significance (*p* < 0.05).

For the Google/Internet option, 296 (59.9%) health sciences students reported that they would use this source if they had questions about vaping devices, whereas 198 (40.1%) indicated that they would not. In the social sciences group, 199 (71.8%) students reported that they would consult Google/Internet in case of doubts, while 78 (28.2%) stated that they would not use this source. The same interpretative approach was applied to the remaining information sources.

### 3.7 Association between e-cigarette level of dependence and self-perception of periodontal health

A strong association was observed between self-reported change in gum coloration and vaping dependence, among health sciences students (*p* < 0.002). This finding suggests that students who reported a higher level of vaping dependence were more likely to perceive changes in gingival color associated with the use of these devices. Although the remaining evaluated categories showed moderate to relatively strong associations, none reached statistical significance.

No statistically significant associations were found between self-perceived periodontal health and vaping dependence among social sciences students across the evaluated categories (*p* > 0.05). While moderate associations were observed for variables such as flossing, gum pain, and change in gum color, these associations were not statistically significant. For the item “bleeding when chewing,” association tests could not be performed due to imbalance in the response categories. Specifically, there were no observations in the “yes” category, resulting in insufficient variability. Because Cramér's V requires at least two categories with observed frequencies to compute a valid measure of association, a meaningful analysis was not possible for this variable.

## 4 Discussion

This study examined sociodemographic characteristics, use and dependence of vaping devices, knowledge about e-cigarettes ingredients, health risks, and self-perception of periodontal health, among university students from health sciences and social sciences careers at the University of Costa Rica. We also assessed if university students consult reputable sources when searching for information regarding e-cigarettes. The prevalence of vaping was 9% among health sciences students and 22% among social sciences students. Regarding dependence, most participants in both groups reported no vaping dependence. Our findings indicate that both health sciences and social sciences students demonstrate gaps in knowledge regarding the composition and potential health effects of vaping devices, although a greater lack of awareness was observed among social sciences students.

In terms of effects in general health, students from both academic areas showed limited understanding of certain effects, particularly neurological outcomes such as increased risk of depression and seizures. Health sciences students demonstrated greater awareness of the oral and systemic health implications of vaping. Additionally, students from both academic areas indicated that they would primarily seek information about vaping through Google/Internet rather than consulting reliable sources such as healthcare professionals. Additionally, more students from the health sciences area that vaped, reported perceiving changes in gingival coloration compared with social sciences students.

In the present study, the mean age of health sciences students who reported vaping was 22.12 ± 2.73 years, while among social sciences students was 20.99 ± 2.73 years. These findings are consistent with international evidence. A study conducted in South Korea among individuals aged 18–30 years reported a similar mean age of 21 years among users (21). In the United States, 11% of individuals aged 18–24 reported current e-cigarette use (occasional or daily) in 2021, with exclusive vaping more common than combustible tobacco or dual use (22). In Europe, regular e-cigarette use among individuals aged ≥15 years was estimated at 1.8% in 2017, representing a 21.2% increase since 2014, and by 2019 current use among those aged 15–24 had reached 5.1% (23, 24). Similarly, data from the Costa Rican Social Security Fund indicate increasing vaping prevalence and related health conditions among individuals aged 15–29 years (25). Collectively, these findings underscore that vaping is increasingly concentrated among younger populations worldwide, including in Costa Rica.

Based on our findings, social sciences students reported a higher prevalence of vaping compared with health sciences students. This difference may suggest that health sciences students are more informed about vaping devices, their recent emergence in the country, and their potential implications for overall health, which could influence lower use within this group. The prevalence observed among social sciences students (22%) is higher than that reported in studies conducted among Palestinian university students (18.1%) (26) and university students in Qatar (14%) (27). Moreover, recent studies focusing exclusively on medical students in Turkey have reported even lower prevalence rates, approximately 4% (28). Other studies assessing the prevalence of e-cigarette use and related knowledge among health sciences students in Trinidad and Tobago have reported usage rates as high as 14% (29). These findings suggest variability in vaping behaviors across academic disciplines and geographical contexts.

Regarding academic year, the present study found that within the health sciences, third- and fifth-year students reported the highest frequency of vaping, whereas sixth-year students reported the lowest frequency. This pattern may be associated with age and level of academic training, as students in more advanced years may possess greater knowledge about the potential health risks of these devices. However, academic year distribution differed significantly between health sciences and social sciences students (*p* < 0.001), which may partially explain differences in knowledge and vaping patterns independent of field of study. In the social sciences, vaping was more frequently reported among first-, second-, and third-year students. Several studies indicate that e-cigarette initiation most commonly occurs during the transition to university, a stage characterized by greater independence, less parental oversight, and stronger peer influence. For instance, among Thai students, 76.2% began using e-cigarettes during their first year of university, whereas 23.8% initiated use in secondary school (30). In Chile, the average age of initiation was 18 years (±2.2), while in a university sample from Qatar it was slightly higher, at 20.4 years (±8.2) (27, 31).

In our study, only a small proportion of participants were classified as highly dependent on vaping devices. However, this finding should be interpreted with caution, as individuals who do not vape daily may perceive themselves as non-dependent, potentially leading to underestimation and gaps in the assessment of dependence. Furthermore, scientific evidence suggests that higher levels of dependence are more commonly observed among individuals who previously smoked conventional tobacco cigarettes daily and subsequently quit. In such cases, former smokers may turn to vaping more frequently and select higher nicotine concentrations, thereby increasing their risk of developing greater dependence (32).

In the present study, students from health sciences careers demonstrated greater knowledge about e-cigarettes and their components compared with students from the social sciences. However, substantial knowledge gaps were identified in both groups. Although volatile organic compounds were more frequently recognized by health sciences students, a notable proportion of participants reported not knowing the ingredients contained in vaping devices or chose not to respond. These findings are consistent with previous research among medical students in Spain, which also reported limited awareness regarding the presence of nicotine, propylene glycol, and glycerin in e-cigarettes (33). The persistence of these misconceptions among university students is concerning because insufficient knowledge about product composition may reduce risk perception and contribute to the normalization of vaping behaviors.

This limited awareness is particularly problematic given the increasing evidence on the potential toxicity of e-cigarette aerosols. The vaping process can generate carbonyl compounds such as acetaldehyde and promote the release of heavy metals, including nickel, cadmium, chromium, and lead, which have been associated with inflammatory responses, cellular damage, and increased susceptibility to oral diseases (34–36). In our study, awareness of specific components such as propylene glycol and heavy metals remained relatively low, especially among social sciences students. This is clinically relevant because propylene glycol may reduce salivary flow and lower salivary pH, thereby weakening salivary defense mechanisms and increasing the risk of dental caries and enamel erosion (37, 38), while exposure to heavy metals has been linked to neurocognitive and systemic health effects (39). In addition, nicotine delivered through e-cigarettes may disrupt the blood–brain barrier and produce higher serum nicotine concentrations than conventional cigarettes, with potential implications for both oral and systemic health (40, 41). Flavoring agents and viscous aerosols have also been shown to alter oral epithelial cells and disrupt commensal oral biofilms, potentially leading to dysbiosis and greater susceptibility to oral diseases (42, 43).

Beyond oral health, vaping may also have broader systemic and neurological implications. Although most participants in this study were aware of commonly reported risks such as pulmonary and cardiovascular diseases, knowledge of less recognized outcomes remained limited. For instance, only 29.8% of health sciences students and 17.7% of social sciences students were aware of the potential association between vaping and seizures, despite evidence indicating that seizures may occur shortly after e-cigarette use (44). Similarly, awareness of the relationship between vaping and depression was limited, even though recent studies suggest a bidirectional association between e-cigarette use and depressive symptoms (45).

Several components of vaping devices have documented effects on oral tissues, suggesting a potential impact on gingival health. In the present study, students from health sciences careers reported a significant association between self-perceived changes in gingival coloration, which may reflect greater awareness of oral health conditions among individuals trained in health-related fields. Experimental evidence indicates that e-cigarette aerosols increase oxidative and carbonyl stress and trigger inflammatory responses in gingival epithelial cells, and chronic exposure may activate molecular pathways associated with inflammation, DNA damage, and cellular senescence, thereby increasing susceptibility to periodontitis (46). Comparative studies among individuals who vape, conventional cigarette smokers, and non-smokers have also reported differences in gingival inflammation and higher volumes of gingival crevicular fluid among vaping groups, possibly related to the vasoconstrictive effects of nicotine and other e-liquid components (47). Similarly, research conducted in adults in South Korea has associated vaping with higher prevalence of periodontal disease and increased levels of inflammatory biomarkers and growth factors in saliva and gingival crevicular fluid (48). Despite these findings, long-term evidence remains limited, and further large-scale studies are needed to better understand the oral health consequences of vaping and its potential role in the development of oral diseases.

Our findings suggest that there may be limited public knowledge about these products, which could influence how individuals intend to seek information. Accordingly, our questionnaire asked participants whom they would consult if they had questions regarding vaping devices, reflecting hypothetical rather than actual behaviors. The distribution of responses was very similar across the two academic areas, indicating that students in both groups may not have a clearly defined or preferred source of consultation and might be inclined to rely on potentially unreliable information channels. Similar patterns have been reported in a study conducted among students at the University of Louisville, where most participants indicated they would consult Google, followed by friends, while only a small proportion reported they would seek advice from vape shop personnel (17). Notably, in our study, a higher proportion of students from the social sciences indicated they would consult healthcare professionals compared with students from the health sciences, despite the expectation that the latter would be more aware that physicians and dentists represent the most reliable sources of evidence-based information regarding vaping devices.

Limitations of our study should be acknowledged. First, a higher proportion of responses were obtained from women compared with men and individuals of other genders, which may introduce selection and voluntary participation bias. This imbalance may also suggest that vaping remains a gender-stigmatized topic, with men potentially reporting higher usage but being less willing to participate in surveys involving sensitive information, despite awareness of the potential health risks associated with these behaviors. Additionally, social desirability bias may have influenced participants’ responses. Because the study was conducted within the context of the University of Costa Rica, some participants may have felt concerned or uncertain about providing fully truthful responses, particularly given the sensitive nature of certain questions. As a result, some responses may reflect what participants perceived as socially acceptable answers rather than their actual behaviors, potentially limiting the interpretability of certain survey items and introducing reporting bias.

Furthermore, although adjusted logistic regression analyses controlling for sex, age, and academic year were subsequently performed, academic year differed significantly between health sciences and social sciences students. Therefore, academic seniority, rather than field of study alone, may still partially explain some of the observed differences in vaping-related knowledge and behaviors.

Another important limitation relates to the adaptation and validation of the questionnaire instruments. Although the survey was based on previously published and validated questionnaires and followed cross-cultural adaptation principles, some sections underwent content modifications to improve their applicability to the target population. In particular, the self-perception of periodontal health questionnaire was adapted by removing items associated with manifestations of moderate-to-severe periodontitis that were considered unlikely in this population of young university students. Although the adapted instrument demonstrated acceptable internal consistency during pilot-testing, formal construct validity analyses, such as factor analysis, were not performed. Additionally, some questionnaire sections included heterogeneous multiple-response items assessing conceptually distinct domains, limiting their interpretation as unidimensional psychometric constructs. Therefore, Cronbach's alpha values should be interpreted as exploratory indicators of internal consistency rather than evidence of full psychometric validation. These limitations may introduce some degree of misclassification and restrict the generalizability and psychometric robustness of the findings.

In addition, several sections of the questionnaire relied primarily on dichotomous (yes/no) response formats, which may have reduced measurement sensitivity and limited the ability to capture gradations in participants’ perceptions, behaviors, and knowledge. Consequently, some degree of misclassification bias cannot be excluded. However, the questionnaire was adapted from previously published instruments that originally used close-ended and dichotomous response formats, particularly for the assessment of self-perceived periodontal health. The use of these formats was intentionally maintained to facilitate comprehension, reduce respondent burden, and improve completion rates in an online university-based survey. Nevertheless, future studies may benefit from incorporating Likert-type or multi-category response scales to improve sensitivity and allow a more nuanced assessment of vaping-related knowledge and perceptions.

Several strengths of this study should be highlighted. First, a representative sample students from the health sciences and social sciences areas was included in the study. Furthermore, the survey was administered at a time when vaping was still a relatively new topic in the country and when information in mass media was limited. As a result, participants’ responses likely reflected their baseline knowledge and perceptions at that moment. Subsequently, public communication campaigns were implemented by the Ministry of Health of Costa Rica to inform the population about these devices. Recently, as mentioned, Costa Rica has strengthened regulatory oversight of vaping products through an executive decree that establishes a binding sanitary framework for vaping liquids, both with and without nicotine. This context provides an opportunity for future longitudinal or follow-up studies using the same instrument to assess the impact of public health policies and official health communication on the knowledge and perceptions of the university population.

## 5 Conclusions

Differences in vaping behaviors between health sciences and social sciences students may partly reflect differences in academic training and exposure to health-related education, which could influence both behavior and risk perception. Importantly, these associations remained significant after adjustment for sex, age, and academic year, suggesting that academic field is independently associated with vaping-related outcomes, although residual confounding by academic seniority cannot be completely excluded.

Knowledge regarding the specific components and health risks of vaping devices was limited in both groups. This finding is particularly relevant for health sciences students, who as future healthcare professionals will play a central role in patient education, risk communication, and prevention strategies related to vaping-associated harms.

Most participants reported low to moderate levels of vaping dependence, although a non-negligible proportion showed higher levels of dependence. Given the addictive potential of nicotine-containing electronic devices, these findings underscore the need for early prevention and intervention strategies within university populations. In this context, clear, evidence-based educational approaches delivered through healthcare professionals and institutional communication channels are warranted.

Overall, the results highlight the need for targeted educational interventions addressing vaping use, health effects, and dependence in university settings. The observed knowledge gaps suggest potential limitations in access to accurate or comprehensive information among students. Strengthening educational strategies and incorporating up-to-date evidence on vaping into health sciences curricula is essential to better prepare future healthcare professionals and to support public health efforts aimed at reducing e-cigarette use among young adults.

## Institutional review board statement

The study was conducted following the Declaration of Helsinki and approved by the Scientific Ethics Committee of the University of Costa Rica (CEC-107-2024), approved March 21, 2024.

## Informed consent statement

Informed consent was obtained from all subjects involved in the study. No identifiable information about participants is included in this publication.

## Data Availability

The original contributions presented in the study are included in the article/Supplementary Material, further inquiries can be directed to the corresponding author/s.
